# Increasing Genetic Testing Uptake Through Workflow Optimization: A Quality Improvement Study in Pediatric Hearing Loss

**DOI:** 10.3390/children13020240

**Published:** 2026-02-09

**Authors:** Jennifer Coto, Julia Anne Morris, Valerie Yunis, Chrisanda Marie Sanchez, Xue Zhong Liu, Ivette Cejas

**Affiliations:** 1 Department of Pediatrics, University of Miami, Miami, FL 33136, USA; jennifercoto@med.miami.edu (J.C.);; 2 Department of Otolaryngology, University of Miami, Miami, FL 33136, USA; 3 Morsani College of Medicine, University of South Florida, Tampa, FL 33620, USA

**Keywords:** hearing loss, pediatrics, genetic testing, quality improvement

## Abstract

**Highlights:**

**What are the main findings?**

- Implementation of a standardized genetics referral protocol more than doubled documented referral rates for children with hearing loss in an otology and audiology clinic.
- Use of predefined referral pathways increased consistency in referral practices across providers and referral modalities.

**What is the implication of the main finding?**

- Structured referral pathways and brief provider education can meaningfully reduce gaps between guideline recommendations and real-world clinical practice.
- Quality improvement strategies offer a scalable approach to improving access to genetic evaluation and advancing precision care for pediatric hearing loss.

**Abstract:**

Background/Objectives: Although genetic testing is recommended for pediatric hearing loss, referral rates within otology and audiology practices remain low. This study evaluated referral rates, referral pathways, and genetics appointment completion before and after implementation of a quality improvement (QI) referral protocol in an ethnically diverse pediatric cohort. Methods: Phase 1 (January–August 2023) included chart reviews of 88 pediatric patients with hearing loss to assess whether genetics referrals were made and completed. Data collected included demographics, referral modality (clinical note documentation, routed note to genetics, or direct referral order), and appointment status. In Phase 2 (September 2023–September 2024), a standardized referral protocol was implemented requiring all newly diagnosed patients to be referred using one of three predefined pathways. Providers received brief training and reminder cards. Chart reviews were then conducted for an additional 114 patients. Results: A total of 202 patients were included (Phase 1: *n* = 88; Phase 2: *n* = 114). Following protocol implementation, the proportion of patients with any documented genetics referral increased significantly (35.2% vs. 68.4%, χ^2^ = 22.03, *p* < 0.001). Referral order placement, documentation, and note routing increased across all referral modalities (all *p* < 0.001). Genetics appointment completion also improved significantly, from 11.4% in Phase 1 to 38.6% in Phase 2 (*p* < 0.001). Conclusions: Genetic referrals for pediatric hearing loss remain underutilized but improved substantially following implementation of a standardized referral protocol. These findings highlight the importance of optimizing referral pathways and providing ongoing provider education. QI initiatives represent a practical strategy to enhance access to genetic evaluation and support precision care.

## 1. Introduction

Hearing loss is the most common sensory disorder, affecting more than 430 million people worldwide, including 34 million children [1]. In developed countries, approximately 1.7 to 3 per 1000 children are born with detectable hearing loss, with prevalence increasing to approximately 3.5 per 1000 by adolescence [2,3]. In pediatric populations, 50–70% of hearing loss is attributable to genetic mutations [4,5]. These may be syndromic (~30%), where hearing loss occurs in combination with other systemic symptoms, such as in Usher, Pendred, Alport, or Jervell and Lange-Nielsen syndromes, or non-syndromic (~70%), with common variants including *GJB2*, *OTOF*, and *TMPRSS3* [5,6]. The burden on affected children and families is substantial. Hearing loss in children contributes to delays in speech and language development, reduced educational outcomes, and social isolation [5,7,8]. Furthermore, approximately 60% of educationally significant hearing loss is caused by genetic factors [6]. Therefore, the current study aimed to assess the impact of implementing a streamlined referral pathway within an otology and audiology practice on rates of genetic testing completion as part of a quality improvement (QI) initiative.

Importantly, children who receive early intervention with hearing amplification or cochlear implants (CI) have been shown to have improved long-term language outcomes [8,9,10]. Delayed diagnosis and intervention can worsen prognosis. Information regarding genetic diagnosis may further inform clinical decision-making by clarifying prognosis, supporting earlier determination of cochlear implant candidacy, and enabling intervention during critical periods of auditory and language neuroplasticity, which has been linked to improved CI outcomes [11], particularly in variants such as *GJB2* or *OTOF*, which are associated with better auditory performance post-implantation than other genetic causes [12].

Given the heterogeneity of hearing loss, involving more than 100 implicated genes and over 400 associated genetic syndromes, genetic testing has become essential to diagnostic evaluation [6]. The American College of Medical Genetics and Genomics (ACMG) recommends genetic testing for all children with hearing loss, citing benefits for clinical management, prognostic accuracy, genetic counseling, intervention planning, and targeted surveillance for syndromic comorbidities [6,13,14]. Other major organizations, including the Joint Committee on Infant Hearing (JCIH), the American Academy of Pediatrics (AAP), the American Speech-Language-Hearing Association (ASHA), and the International Pediatric Otolaryngology Group (IPOG), also endorse genetic testing for infants with hearing loss [14]. Beyond medical management, genetic testing provides critical counseling information by clarifying etiology, recurrence risk, and intervention options [4,5,15,16].

Genetic testing also enables early identification and monitoring of syndromic complications, such as progressive vision loss in Usher syndrome, kidney function in Alport syndrome, thyroid function in Pendred syndrome, or potentially life-threatening arrhythmias caused by QT prolongation in Jervell and Lange-Nielsen syndrome [5,13,16]. It is well established that genetic screening as early as possible is most effective in optimizing outcomes in these patients [5,15]. Advances in genetic testing technology, particularly the transition from Sanger sequencing to next-generation sequencing (NGS), have substantially improved access to comprehensive testing. NGS enables the simultaneous analysis of thousands of genes at a cost comparable to traditional single-gene approaches [16]. This expanded capacity is especially important for hearing loss, which is highly genetically heterogeneous, as broader panel-based testing increases diagnostic yield compared with targeted testing strategies [16,17].

Despite established professional guidelines and well-documented benefits of etiologic and genetic evaluation, genetic testing for pediatric hearing loss remains underutilized. Recent surveys indicate that fewer than half of providers routinely refer children with hearing loss for genetic testing, and many families are never offered testing at all [14,18]. Reported barriers include limited provider awareness, inconsistent referral practices, workflow inefficiencies, restricted access to genetic counseling, and insurance or cost-related concerns. Notably, these gaps persist even in well-resourced settings. At our large, multi-ethnic, university-based hospital system with an established multidisciplinary team that includes on-site genetics services, baseline referral rates remained suboptimal, suggesting that system-level workflow barriers—rather than resource availability alone—may impede appropriate testing.

To address this gap, we implemented a quality improvement (QI) initiative designed to standardize and streamline the genetics referral process within our otology and audiology practice. The primary aim was to increase the proportion of children with hearing loss referred for genetic evaluation by embedding a structured referral workflow into routine clinical care. To assess the impact of this intervention, we conducted a retrospective chart review comparing referral completion and testing outcomes before and after protocol implementation.

## 2. Materials and Methods

### 2.1. Procedures

As part of a QI initiative, a retrospective chart review was completed for patients seen at the Children’s Hearing Program (CHP) at the University of Miami for patients under the age of 17 who had received a diagnosis of hearing loss from January 2023–August 2023 (Phase 1) and September 2023 to September 2024 (Phase 2). These two phases represented pre- and post-implementation periods for the quality improvement intervention. Phase 1 reflected usual care referral practices prior to protocol implementation, whereas Phase 2 captured outcomes after introduction of the standardized referral workflow, allowing comparison of referral rates before and after the intervention. Assignment to Phase 1 or Phase 2 was based solely on the date of the patient’s diagnostic visit relative to the timing of protocol implementation. Hearing loss criteria included children with diagnosed unilateral or bilateral sensorineural (SNHL) mixed (MHL), or conductive (CHL) hearing loss. For the CHL inclusion, children were included if they were considered to have long-term or chronic CHL (e.g., microtia/atresia and other middle ear anomalies such as cholesteatoma or ossicular malformations). Children with transient otitis media were not referred for genetic evaluation. Exclusion criteria included patients who presented to clinic for reimplant or had explant surgeries and those who had undergone genetic testing elsewhere. Demographic variables extracted for each patient included demographic information (age, sex, ethnicity, race, language) along with medical information (insurance type, hearing loss degree, and device(s) used).

During Phase I, variables collected during the chart review included if a referral order for genetic testing was placed, if the chart was routed to the genetics team, and if any mention of a referral to genetics was made in the clinical note. For each of these variables, we also indicated which provider (i.e., otologist, audiologist, etc.) completed the task. Additionally, we extracted whether a genetics appointment was made, the status of the appointment (completed, no-show, or canceled), and whether a genetic mutation was identified.

After completion of Phase 1 data collection, a QI protocol was implemented to increase referral rates to genetics for patients newly diagnosed with hearing loss. The protocol introduced three standardized referral pathways that were applied to all patients at the time of diagnosis. Specifically, during the diagnostic appointment, providers were instructed to: (1) document the genetics referral in the recommendations section of the clinical note; (2) route the clinical note to the genetics team; and (3) place an electronic referral order for genetic testing.

To support implementation, audiologists and otologists received email communication outlining the updated referral procedures. The updated procedures were also discussed during a monthly department meeting and sufficient time was allocated for any questions by providers. In addition, laminated ID badge cards with simplified step-by-step instructions were distributed to all providers, and the same instructions were posted at clinic workstations for point-of-care reference. Finally, an electronic medical record (EMR) smart phrase was developed to facilitate consistent documentation of genetics referrals within clinical notes. After the protocol was implemented, we conducted Phase 2 chart reviews to extract the same variables as Phase 1.

### 2.2. Data Analysis

Descriptive statistics were used to summarize demographic variables as well as referral characteristics for Phase 1 and Phase 2 cohorts. Continuous variables were summarized using medians and interquartile range (IQR) and compared using the Mann-Whitney U test. Categorical variables were summarized using frequencies and percentages and compared using chi-square tests or Fisher’s exact tests when expected cell counts were small. All chart review variables were complete with no missing data. Some demographic variables contained missing values; these were reported using available-case denominators in the demographic table (n and %) to reflect the number of participants with available data. Because demographic variables were not included in inferential analyses, no imputation or case exclusion procedures were necessary.

## 3. Results

### 3.1. Demographics

Chart reviews were completed on two hundred and two patients (Phase 1 *n* = 88; Phase 2 *n* = 114). Patients in Phase 1 were older on average (median =  72 months, IQR = 84.0) than those in Phase 2 (median = 54 months, IQR = 74.75), with a Mann–Whitney U test indicating a significant difference between groups (U = 5868.5, *p* = 0.038). See Table 1 for sample characteristics.

Approximately half of the sample was female across both phases (Phase 1: 52.3%; Phase 2: 51.8%). The racial composition was predominantly White (Phase 1: 69.9%; Phase 2: 75.7%), followed by African American patients (Phase 1: 21.7%; Phase 2: 13.1%). Further, a majority of participants identified as Hispanic (Phase 1: 55.6%; Phase 2: 73.1%).

English was the most frequently reported primary language in both phases (Phase 1: 73.6%; Phase 2: 65.5%), followed by Spanish (Phase 1: 25.3%; Phase 2: 31.9%). Public insurance was the most common insurance type in Phase 1 (52.3%), whereas private insurance was slightly more prevalent in Phase 2 (50.9%).

Regarding hearing devices, the most common device type in both phases was bilateral HA (Phase 1: 36.4%; Phase 2: 46.0%). Full demographic and clinical characteristics by phase are presented in Table 1.

### 3.2. Genetics Referral Outcomes by Phase

#### 3.2.1. Referred to Genetics via Any Method

The proportion of patients with evidence of any genetics referral method differed significantly between phases. In Phase 1, 35.2% of patients had documentation of a genetics referral by any method, compared to 68.4% of patients in Phase 2. A chi-square test indicated this difference was statistically significant, χ^2^(1, *N* = 202) = 22.03, *p* < 0.001. The odds of having any genetics referral were significantly higher in Phase 2 relative to Phase 1 (OR = 3.98, 95% CI [2.21, 7.18]).

#### 3.2.2. Referrals by Method

Significant phase differences were also observed across different methods of the genetics referral process. Specifically, whether a referral order was placed differed by phase, χ^2^(3, *N* = 202) = 32.36, *p* < 0.001. In Phase 1, 18.2% of patients had a referral order placed, compared to 46.5% in Phase 2. Similarly for documentation of a referral in the clinical note, this varied by phase with documentation present for 34.1% of Phase 1 patients versus 64.0% of Phase 2 patients [χ^2^(3, *N* = 202) = 36.89, *p* < 0.001]. Similarly, routing of clinical notes to the genetics team increased significantly from Phase 1 to Phase 2, χ^2^(3, *N* = 202) = 32.71, *p* < 0.001, with 5.7% of Phase 1 notes routed compared to 34.2% in Phase 2.

#### 3.2.3. Referral Initiator

For referral order placement, the distribution of referral initiators varied by phase, χ^2^(3, *N* = 202) = 32.36, *p* < 0.001. In Phase 1, referral orders were placed exclusively by ENT providers (18.2%), with no orders initiated by audiologists or jointly. In contrast, Phase 2 demonstrated a more distributed referral pattern, with orders placed by audiologists alone (23.7%), ENT providers alone (16.7%), and jointly by audiologists and ENT/APRN providers (6.1%).

Similarly, documentation of genetics referrals in the clinical note differed significantly by phase, χ^2^(3, *N* = 202) = 36.89, *p* < 0.001. In Phase 1, documentation was most commonly completed by ENT providers (18.2%), followed by audiologists (9.1%) and joint documentation (6.8%). In Phase 2, documentation was more frequently completed by audiologists (43.0%), with additional documentation by ENT providers (7.0%) and jointly by both provider types (14.0%).

A comparable pattern was observed for the routing of clinical notes to the genetics team, χ^2^(3, *N* = 202) = 32.71, *p* < 0.001. In Phase 1, routed notes were initiated only by ENT providers (5.7%). In Phase 2, note routing occurred primarily through audiologists (28.1%), with additional contributions from ENT providers (3.5%) and joint routing by both provider types (2.6%).

### 3.3. Genetics Appointment Completion and Testing Outcomes

Genetics appointment outcomes differed significantly by phase [χ^2^(3, *N* = 202) = 39.79, *p* < 0.001]. In Phase 1, 11.4% of patients completed a genetics appointment, whereas 38.6% of patients in Phase 2 completed an appointment.

Additionally, number of patients identified with a genetic mutation were significantly different between phases with 4.6% of patients identified in Phase 1 compared to 28.2% of patients in Phase 2 [χ^2^(3, *N* = 201) = 34.92, *p* < 0.001].

## 4. Discussion

In this QI initiative, implementation of a standardized, clinic-wide genetics referral protocol was associated with significant improvements across multiple points in the referral pathway, including documentation, referral order placement, appointment completion, and identification of genetic etiologies for hearing loss.

Within our large, multi-ethnic university-based multidisciplinary audiology and otolaryngology practice, with integrated genetic support, the proportion of patients with a documented genetics referral nearly doubled, completion of a genetics appointment increased more than threefold, and identification of genetic etiology for hearing loss increased significantly. Collectively, these findings suggest that targeted workflow standardization can significantly improve access to and completion of genetic evaluation in pediatric hearing loss without requiring additional institutional resources.

Genetic testing plays a critical role in the evaluation of pediatric hearing loss by informing etiology, prognosis, genetic counseling, and timing of intervention, all of which directly influence clinical outcomes. Identification of a genetic etiology can guide anticipatory guidance, surveillance, and multidisciplinary care for associated syndromic conditions, including Usher syndrome, Pendred syndrome, Alport syndrome, and Jervell and Lange-Nielsen syndrome. Improving access to genetic evaluation may reduce diagnostic uncertainty, support family counseling regarding recurrence risk, and improve coordination of care across specialties. 

This initiative aligns with core QI principles by addressing workflow barriers through standardized process redesign and iterative provider education. Prior to implementation, variability in referral practices likely reflected uncertainty regarding responsibility, timing, and expectations. The QI initiative appears to have improved outcomes by clarifying the referral pathway and reducing reliance on individual provider discretion. Audiologists demonstrated the greatest improvement in referral initiation and documentation, which is clinically important given that they are often the first providers to confirm permanent hearing loss and initiate diagnostic evaluation. Empowering audiologists to initiate and document genetics referrals reduced dependence on downstream provider action and facilitated earlier engagement of genetics services. These findings underscore the importance of shared responsibility within multidisciplinary hearing loss care teams and highlight audiologists as key drivers of timely, guideline concordant genetic evaluation.

This intervention was designed to be sustainable within routine clinical practice. The use of EMR smart phrases, point-of-care reminders, and standardized documentation requires minimal ongoing maintenance and does not rely on additional personnel or clinic time. Implementation in a racially, ethnically, and linguistically diverse pediatric population also suggests that standardized workflows may promote more equitable access to genetic services by mitigating variability related to language barriers, insurance status, or differences in provider counseling practices. These features support long-term sustainability and facilitate adoption of similar workflows in other pediatric audiology and otolaryngology settings.

A modest difference in mean age was observed between Phase 1 and Phase 2, with children in Phase 2 being somewhat younger on average. Although age could theoretically influence referral practices, current pediatric hearing loss guidelines recommend etiologic and genetic evaluation for all children with permanent hearing loss irrespective of age at diagnosis. Therefore, this difference was unlikely to fully explain the observed increase in referrals following protocol implementation.

Prior research consistently demonstrates gaps between guideline recommendations for genetic evaluation and actual clinical practice in pediatric hearing loss. Both the American College of Medical Genetics and Genomics (ACMG) and the Joint Committee on Infant Hearing (JCIH) recommend genetic evaluation as part of the diagnostic workup for children with permanent hearing loss to improve etiologic diagnosis, counseling, and management [6,19]. Despite these recommendations, multiple studies have documented low rates of genetics referral and testing in routine care [20]. QI initiatives in other clinical populations have demonstrated that structured workflow interventions, such as standardized referral tools, provider education, and integration of genetics services into clinical workflows, can significantly improve referral and completion rates for genetic counseling and testing [21,22]. In contrast to resource-intensive models, the standardized referral workflow implemented in the present initiative improved referral and completion outcomes without requiring additional specialized resources. 

Limitations of this study should be acknowledged. This study used a retrospective, non-randomized pre–post design as part of a quality improvement initiative; therefore, causal inferences regarding the effect of the intervention should be interpreted cautiously, as system-level factors may also have influenced outcomes. Although referral outcomes were defined a priori and reflected distinct steps in the clinical workflow, multiple related comparisons were conducted without formal adjustment for multiplicity, consistent with the descriptive and process-focused nature of QI evaluations. In addition, this was a retrospective, single-institution initiative, which may limit generalizability. Genetic testing performed outside our institution was excluded to maintain focus on referral practices within our clinical workflow; this exclusion may result in underestimation of overall genetic testing rates. Additionally, retrospective chart review is inherently limited by incomplete documentation. Nevertheless, improving documentation accuracy remains a critical component of effective care coordination and continuity. Finally, the study timeframe limited assessment of long-term patient outcomes, including the downstream clinical impact of earlier genetic diagnosis.

## 5. Conclusions

Implementation of a standardized referral protocol for genetic evaluation in pediatric hearing loss significantly improved referral rates, the completion of genetic appointments, and the identification of genetic etiologies in our cohort. This QI initiative demonstrates that low-cost, standardized workflow implementation can significantly improve adherence to guideline recommended care for pediatric hearing loss. The adoption of similar referral standardization and provider training may help close the gap between evidence-based recommendations for genetic testing and clinical practice in pediatric ENT care. Future work should evaluate the sustainability of this intervention over time, assess patient-centered outcomes, and explore implementation across diverse clinical settings to further advance equitable, guideline concordant care for children with hearing loss.

## Figures and Tables

**Table 1:** Sample Characteristics by study phase (pre- and post-QI implementation).

	Phase 1 (Pre-QI) *n* = 88	Phase 2 (Post-QI) *n* = 114
Characteristic	Median (*IQR*)	Median (*IQR*)
Child Age (months)	72 (84)	54 (74.75)
**Characteristic**	***n* (%)**	***n* (%) **
Child sex Female Male	46 (52.3%) 42 (47.7%)	59 (51.8%) 55 (48.2%)
Child race White Black Asian More than one race	58 (69.9%) 18 (21.7%) 3 (3.6%) 4 (4.8%)	81 (75.7%) 14 (13.1%) 2 (1.9%) 10 (9.3%)
Child ethnicity Hispanic Non-Hispanic	40 (55.6%) 32 (44.4%)	68 (73.1%) 25 (26.9%)
Devices used None Hearing Aid(HA), unilateral HA, bilateral CI and HA, Bimodal CI, unilateral CI, bilateral Bone Conduction Device (BCD), unilateral BCD, bilateral	19 (21.6%) 15 (17%) 32 (36.4%) 1 (1.1%) 0 (0%) 6 (6.8%) 11 (12.5%) 4 (4.5%)	17 (15%) 14 (12.4%) 52 (46%) 7 (6.2%) 2 (1.8%) 7 (6.2%) 12 (10.6%) 2 (1.8%)
Primary language English Spanish Other	64 (73.6%) 22 (25.3%) 1 (1.1%)	74 (65.5%) 36 (31.9%) 3 (2.7%)
Insurance type None Private Public	6 (6.8%) 36 (40.9%) 46 (52.3%)	3 (2.6%) 58 (50.9%) 53 (46.5%)

Note. HA = hearing aid, CI = cochlear implant, BCD = bone conduction device.

## Data Availability

The original contributions presented in this study are included in the article. Further inquiries can be directed to the corresponding author.

## References

[1] World Health Organization Deafness and Hearing Loss. https://www.who.int/news-room/fact-sheets/detail/deafness-and-hearing-loss.

[2] Korver A.M.H., Smith R.J.H., Van Camp G., Schleiss M.R., Bitner-Glindzicz M.A.K., Lustig L.R., Usami S., Boudewyns A.N. (2017). Congenital Hearing Loss. Nat. Rev. Dis. Primers.

[3] National Institute on Deafness and Other Communication Disorders (NIDCD) Quick Statistics About Hearing, Balance, & Dizziness. https://www.nidcd.nih.gov/health/statistics/quick-statistics-hearing.

[4] Lee N.K., Uhler K.M., Yoon P.J., Santos-Cortez R.L.P. (2024). Clinical Genetic Testing for Hearing Loss: Implications for Genetic Counseling and Gene-Based Therapies. Biomedicines.

[5] Božanić Urbančič N., Battelino S., Tesovnik T., Trebušak Podkrajšek K. (2020). The Importance of Early Genetic Diagnostics of Hearing Loss in Children. Medicina.

[6] Alford R.L., Arnos K.S., Fox M., Lin J.W., Palmer C.G., Pandya A., Rehm H.L., Robin N.H., Scott D.A., Yoshinaga-Itano C. (2014). American College of Medical Genetics and Genomics Guideline for the Clinical Evaluation and Etiologic Diagnosis of Hearing Loss. Genet. Med..

[7] Zhong L.X., Kun S., Jing Q., Jing C., Denise Y. (2013). Non-Syndromic Hearing Loss and High-Throughput Strategies to Decipher Its Genetic Heterogeneity. J. Otol..

[8] Moeller M.P., Tomblin J.B., Yoshinaga-Itano C., Connor C.M., Jerger S. (2007). Current State of Knowledge: Language and Literacy of Children with Hearing Impairment. Ear Hear..

[9] Ching T.Y.C. (2015). Is Early Intervention Effective in Improving Spoken Language Outcomes of Children With Congenital Hearing Loss?. Am. J. Audiol..

[10] Stika C.J., Eisenberg L.S., Johnson K.C., Henning S.C., Colson B.G., Ganguly D.H., DesJardin J.L. (2015). Developmental Outcomes of Early-Identified Children Who Are Hard of Hearing at 12 to 18months of Age. Early Hum. Dev..

[11] Han J.H., Kim S.H., Moon I.S., Joo S.Y., Kim J.A., Gee H.Y., Jung J., Choi J.Y. (2023). Comprehensive Prediction Model, Including Genetic Testing, for the Outcomes of Cochlear Implantation. Ear Hear..

[12] Ismail N.M., Galal S.B., Behairy R.M., Sabry R.M. (2024). Systematic Review of Outcomes of Cochlear Implantation of Different Genotypes in Patients with Auditory Neuropathy Spectrum Disorder. Egypt. J. Otolaryngol..

[13] Xiang J., Jin Y., Song N., Chen S., Shen J., Xie W., Sun X., Peng Z., Sun Y. (2022). Comprehensive Genetic Testing Improves the Clinical Diagnosis and Medical Management of Pediatric Patients with Isolated Hearing Loss. BMC Med. Genom..

[14] Barnett C.L., Malhotra P., VanHorn A., Zaharieva B., Myers J., Riggs W.J., Jordan E. (2025). Utilization of Genetics Services in the Diagnosis of Hearing Loss in Newborns in the State of Ohio. J. Community Genet..

[15] Robin N.H., Prucka S.K., Woolley A.L., Smith R.J. (2005). The Use of Genetic Testing in the Evaluation of Hearing Impairment in a Child. Curr. Opin. Pediatr..

[16] Gooch C., Rudy N., Smith R.J.H., Robin N.H. (2021). Genetic Testing Hearing Loss: The Challenge of Non Syndromic Mimics. Int. J. Pediatr. Otorhinolaryngol..

[17] Yan D., Tekin M., Blanton S.H., Liu X.Z. (2013). Next-Generation Sequencing in Genetic Hearing Loss. Genet. Test. Mol. Biomark..

[18] Cejas I., Coto J., Sarangoulis C.M., Yunis V., Blanton S., Liu X.Z. (2025). Parent Experiences with Genetic Testing for Pediatric Hearing Loss. J. Genet. Couns..

[19] Joint Committee on Infant Hearing (2019). Year 2019 Position Statement: Principles and Guidelines for Early Hearing Detection and Intervention Programs. J. Early Hear. Detect. Interv..

[20] Bush M.L., Hardin B., Rayle C., Lester C., Studts C.R., Shinn J.B. (2015). Rural Barriers to Early Diagnosis and Treatment of Infant Hearing Loss in Appalachia. Otol. Neurotol..

[21] Plaskocinska I., Shipman H., Drummond J., Thompson E., Buchanan V., Newcombe B., Hodgkin C., Barter E., Ridley P., Ng R. (2016). New Paradigms for BRCA1/BRCA2 Testing in Women with Ovarian Cancer: Results of the Genetic Testing in Epithelial Ovarian Cancer (GTEOC) Study. J. Med. Genet..

[22] Swanson C.L., Kumar A., Maharaj J.M., Kemppainen J.L., Thomas B.C., Weinhold M.R., Slaby K.M., Mara K.C., Wick M.J., Bakkum-Gamez J.N. (2018). Increasing Genetic Counseling Referral Rates through Bundled Interventions after Ovarian Cancer Diagnosis. Gynecol. Oncol..

